# Mechanistic Model for the Coexistence of Nitrogen Fixation and Photosynthesis in Marine *Trichodesmium*

**DOI:** 10.1128/mSystems.00210-19

**Published:** 2019-08-06

**Authors:** Keisuke Inomura, Samuel T. Wilson, Curtis Deutsch

**Affiliations:** a School of Oceanography, University of Washington, Seattle, Washington, USA; b Daniel K. Inouye Center for Microbial Oceanography: Research and Education (C-MORE), University of Hawai’i at Manoa, Honolulu, Hawaii, USA; University of Chicago

**Keywords:** *Trichodesmium*, carbon, nitrogen, nitrogen fixation, nitrogenase, oxygen, oxygen barrier, photosynthesis, respiration, respiratory protection

## Abstract

The cyanobacterium *Trichodesmium* is an important contributor of new nitrogen (N) to the surface ocean, but its strategies for protecting the nitrogenase enzyme from inhibition by oxygen (O_2_) remain poorly understood. We present a dynamic physiological model to evaluate hypothesized conditions that would allow *Trichodesmium* to carry out its two conflicting metabolic processes of N_2_ fixation and photosynthesis. First, the model indicates that managing cellular O_2_ to permit N_2_ fixation requires high rates of respiratory O_2_ consumption. The energetic cost amounts to ∼80% of daily C fixation, comparable to the observed diminution of the growth rate of *Trichodesmium* relative to other phytoplankton. Second, by forming a trichome of connected cells, *Trichodesmium* can segregate N_2_ fixation from photosynthesis. The transfer of stored C to N-fixing cells fuels the respiratory O_2_ consumption that protects nitrogenase, while the reciprocal transfer of newly fixed N to C-fixing cells supports cellular growth. Third, despite *Trichodesmium* lacking the structural barrier found in heterocystous species, the model predicts low diffusivity of cell membranes, a function that may be explained by the presence of Gram-negative membrane, production of extracellular polysaccharide substances (EPS), and “buffer cells” that intervene between N_2_-fixing and photosynthetic cells. Our results suggest that all three factors—respiratory protection, trichome formation, and diffusion barriers—represent essential strategies that, despite their energetic costs, facilitate the growth of *Trichodesmium* in the oligotrophic aerobic ocean and permit it to be a major source of new reactive nitrogen.

**IMPORTANCE** *Trichodesmium* is a major nitrogen-fixing cyanobacterium and exerts a significant influence on the oceanic nitrogen cycle. It is also a widely used model organism in laboratory studies. Since the nitrogen-fixing enzyme nitrogenase is extremely sensitive to oxygen, how these surface-dwelling plankton manage the two conflicting processes of nitrogen fixation and photosynthesis has been a long-standing question. In this study, we developed a simple model of metabolic fluxes of *Trichodesmium* capturing observed daily cycles of photosynthesis, nitrogen fixation, and boundary layer oxygen concentrations. The model suggests that forming a chain of cells for spatially segregating nitrogen fixation and photosynthesis is essential but not sufficient. It also requires a barrier against oxygen diffusion and high rates of oxygen scavenging by respiration. Finally, the model indicates that the life span of intracellular oxygen is extremely short, thus enabling cells to instantly create a low-oxygen environment upon deactivation of photosynthesis.

## Abstract (precis)

*Trichodesmium* is a major nitrogen-fixing cyanobacterium and exerts a significant influence on the oceanic nitrogen cycle. It is also a widely used model organism in laboratory studies. Since the nitrogen-fixing enzyme nitrogenase is extremely sensitive to oxygen, how these surface-dwelling plankton manage the two conflicting processes of nitrogen fixation and photosynthesis has been a long-standing question. In this study, we developed a simple model of metabolic fluxes of *Trichodesmium* capturing observed daily cycles of photosynthesis, nitrogen fixation, and boundary layer oxygen concentrations. The model suggests that forming a chain of cells for spatially segregating nitrogen fixation and photosynthesis is essential but not sufficient. It also requires a barrier against oxygen diffusion and high rates of oxygen scavenging by respiration. Finally, the model indicates that the life span of intracellular oxygen is extremely short, thus enabling cells to instantly create a low-oxygen environment upon deactivation of photosynthesis.

## INTRODUCTION

Biological dinitrogen (N_2_) fixation provides bioavailable nitrogen (N) to the marine biosphere, supporting up to half of net community production in otherwise nutrient-depleted environments (1). The process of N_2_ fixation by the enzyme nitrogenase requires large amounts of energy and electrons (2–4). Furthermore, the nitrogenase enzyme contains metal cofactors that are irreversibly disabled in the presence of even trace levels of oxygen (O_2_) (5, 6). The mechanisms by which cells of a few micrometers in size maintain an active nitrogenase enzyme in an O_2_-rich environment are diverse and not fully understood (7). Some nitrogen fixers form a thick glycolipid layer of specialized cells (heterocysts) that prevent O_2_ diffusion into the N_2_-fixing cells (8). Other nitrogen fixers maintain high respiration rates to counteract the passive O_2_ diffusion (9–12). The metabolic strategies that enable N_2_ fixation to function in an oxygenated environment occur at the expense of other physiological activities, including growth. The growth rate handicap of diazotrophs is considered a key ecological trade-off (13–17) with important implications for the global N cycle (18, 19).

In the marine environment, a major contributor to N_2_ fixation is the photosynthetic diazotroph *Trichodesmium*, mainly observed in oligotrophic tropical and subtropical oceans (20–23). Although this species forms trichomes, they do not contain heterocysts to protect from O_2_ invasion (24, 25). Moreover, they are observed to fix N_2_ during the day, when photosynthetic production of O_2_ is also occurring (26, 27). Some studies show *Trichodesmium* respiration rates exceeding those of non-N_2_-fixing cyanobacteria (10, 26), despite a positive net O_2_ evolution rate during the daytime (25). Despite over a century of research on *Trichodesmium*, there is no unequivocal explanation for how N_2_ fixation occurs when the cells are photosynthetically active and O_2_ should be at its highest levels.

In response to this physiological enigma, it has been hypothesized that N_2_ fixation and photosynthesis are temporally and/or spatially segregated (28, 29). Spatial segregation is a highly debated strategy, as some previous work revealed the presence of nitrogenase in almost all cells (29, 30), while other reports showed nitrogenase occurred in about 10% to 20% of the cells (24, 31, 32). In support of temporal segregation, it has been shown that the rates of N_2_ fixation and respiration increase, while the rate of photosynthesis decreases, during the middle of the light period (26). Whether this temporal segregation is sufficient for photosynthesis and N_2_ fixation to occur simultaneously remains unclear (25). A recent approach to the *Trichodesmium* paradox has been to track ^13^C and ^15^N uptake at the cellular level using high-resolution secondary ion mass spectrometry (33, 34). However, even with near-hourly resolution measurements, it was not possible to determine spatial segregation along the trichome because the redistribution of newly fixed N occurs on a time scale of minutes (33).

**FIG 1:**
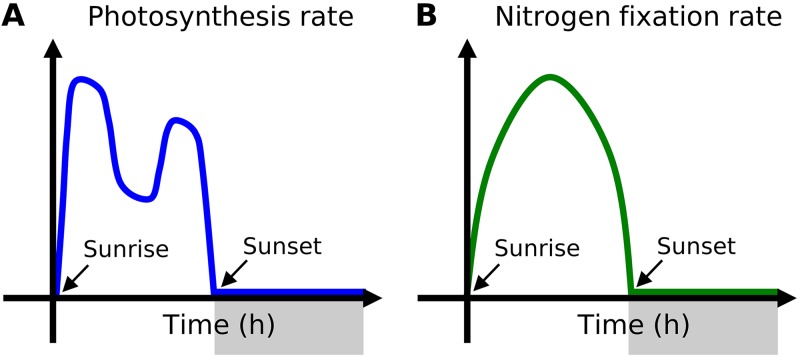
Schematic illustration of observed diurnal cycle of rates of (A) photosynthesis and (B) N_2_ fixation. Gray shading in *x* axes indicates dark periods. Time 0 indicates sunrise. These schematics represent observed general trends (26, 27, 33, 65–70).

Compiling previous studies reveals a common general feature of diurnal cycles in *Trichodesmium* physiology (Fig. 1). Rates of photosynthesis increase at sunrise and peak in early morning (Fig. 1A). The rate decreases during midday, increases slightly again toward evening, and decreases to nearly zero at night. The rate of N_2_ fixation, on the other hand, reaches its maximum value during the midday and its minimum (approximately zero) during the night (Fig. 1B). Similarly to N_2_ fixation, respiration rates peak during the midday, yielding a dip in near-cell O_2_ within a colony. Whereas some studies show reduced O_2_ within a colony of *Trichodesmium* (28), recent work shows that photosynthesis causes the boundary layer to have O_2_ levels that are 20% to 30% higher than those seen in the ambient water, where O_2_ is nearly saturated (34). Therefore, *Trichodesmium* needs to manage O_2_ fluxes not only directly from the photosynthetic cells (P cells) but also from the boundary layer environment within a colony. The question remains as to how these observed trends relate to the temporal coexistence of photosynthesis and N_2_ fixation.

In this study, we take a fresh approach to investigation of the *Trichodesmium* paradox by modeling the physiology of *Trichodesmium* (cell flux model of *Trichodesmium* [CFM-Tricho]) over a diurnal cycle to evaluate the hypothesized spatial and temporal strategies of the cyanobacteria for maintaining N_2_ fixation and photosynthesis. It has been suggested that colony formation by *Trichodesmium* plays an important role in creating a low-O_2_ environment to facilitate N_2_ fixation (28, 35), though some evidence contradicts assertions of such a role (34, 36). Recent studies show that the majority of *Trichodesmium* exists as filaments (37), which have higher rates of N_2_ fixation per cell than colony-maintained *Trichodesmium* cells (38). We focused the model on a single trichome due to its simple morphology. However, the considerations are equally valid for colonies, as the model resolves the near-cell environment (referred to here as the “boundary layer”) where O_2_ concentrations are influenced by cellular metabolism. The model simulates cellular resource allocation by combining a model of the cellular reserves of C and N (39) with a representation of O_2_ management critical to nitrogenase activity (12). The primary mechanisms of O_2_ protection include high respiration rates (respiratory protection [including dark respiration and light-dependent respiration]), segregation of N_2_-fixing cells from photosynthetic cells (trichome formation), and low diffusivity between cells (diffusion barriers). The diurnal variation of metabolism can be explained by fluctuations in the relative abundances of photosynthetic cells and nonphotosynthetic cells. The quantitative model requirements are evaluated against our current knowledge of *Trichodesmium* and other diazotrophs.

## RESULTS

### Simulating cellular differentiation.

**FIG 2:**
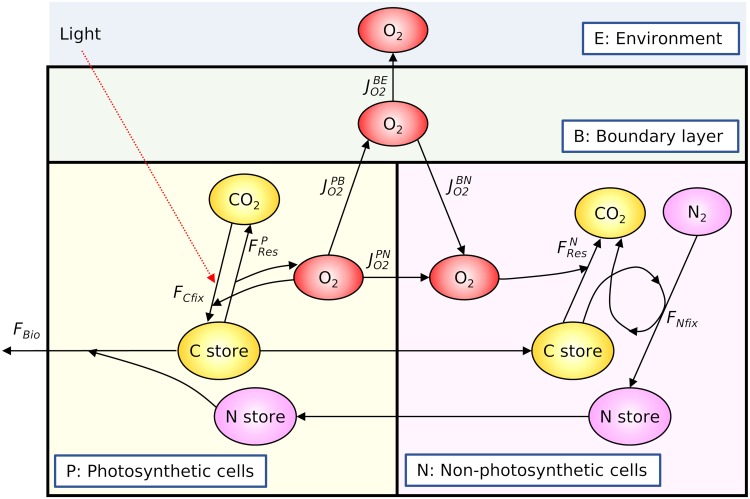
Schematic depiction of molecular pools and fluxes in the model. The model consists of the following four boxes: photosynthetic cells (P), nonphotosynthetic cells (N), boundary layer (B), and environment (E). In each cell type, the model solves for the concentration of stored C and N and the concentration of O_2_. The model assumes that the supply of CO_2_ and N_2_ does not limit the rates of photosynthesis and N_2_ fixation, the assumption made in most ecological models. Each flux symbol corresponds to those used in the model equations (see Materials and Methods).

The model resolves two types of cells: photosynthetic cells (P cells) and nonphotosynthetic cells (N cells) (Fig. 2). The fractions of P and N cells are represented by *f_P_* and *f_N_*, respectively. The P cells fix carbon and make it available for growth, storage, and respiration. The N cells use stored C obtained from P cells for O_2_ consumption and N_2_ fixation. The proportion of cells carrying out each metabolic function determines the rates within and fluxes from the trichome as a whole. We are interested in the rates of N_2_ fixation (*F*_Nfix_; here, “*F*” indicates fluxes), which we assume depend on O_2_ and the standing stocks of stored N and C, as represented in equation 1:

(1) $$F_{\text{Nfix}}=F_{\text{Nfix}}^{\text{max}}\text{ max}\left(\frac{{\left[{\text{O}}_{2}\right]}_{\text{crit}}-{\left[{\text{O}}_{2}\right]}_{N}}{{\left[{\text{O}}_{2}\right]}_{\text{crit}}},\;0\right)\left(\frac{C_{\text{Sto}}^{N}}{C_{\text{Sto}}^{N}+K_{C}}\right)\left(\frac{N_{\text{Sto}}^{\text{max}}-N_{\text{Sto}}}{N_{\text{Sto}}^{\text{max}}}\right)$$

Here, $F_{\text{Nfix}}^{\text{max}}$ is the maximum possible rate of N_2_ fixation for the average cell in the colony, which depends on the fraction of cells with active nitrogenase: $F_{\text{Nfix}}^{\text{max}}$=$F_{\text{Nfix}}^{\text{full}}f_{N}^{}f_{\mathrm{NITROGE}}^{}$ (see Materials and Methods). The remaining terms, which scale that rate between 0 and 1, represent the inhibition by O_2_ in N cells, limitation by C storage in those cells, and inhibition by an excess of stored N in the entire trichome. Complete inhibition occurs for O_2_ in P cells above a critical concentration, [O_2_]_crit_, but declines linearly as O_2_ levels fall below that level. Similarly, N_2_ fixation rates rise as N storage is depleted below a specific value ($N_{\text{Sto}}^{\text{max}}$). Finally, rates of N_2_ fixation increase but saturate with available C storage in the nonphotosynthetic cells ($C_{\text{Sto}}^{N}=C_{\text{Sto}}/f_{N}^{}$).

To resolve the variation in O_2_ concentrations outside the cells due to photosynthesis and respiration, the O_2_ balance in the boundary layer environment is included. The model normalizes fluxes and molecules to the volume of the entire trichome. Therefore, the model can represent any number of cells with a certain proportion of N_2_-fixing cells. The model assumes that the supply of CO_2_ and N_2_ does not limit the rate of photosynthesis and N_2_ fixation, a common assumption of most diazotroph models. Instead, photosynthesis is a function of light and stored C, and N_2_ fixation is limited by stored C and N (equation 1). Further details are provided in Materials and Methods and Text S1 in the supplemental material.

10.1128/mSystems.00210-19.1 TEXT S1 Supplemental methods. Download Text S1, PDF file, 0.2 MB. Copyright © 2019 Inomura et al. 2019 Inomura et al. This content is distributed under the terms of the Creative Commons Attribution 4.0 International license.

**FIG 3:**
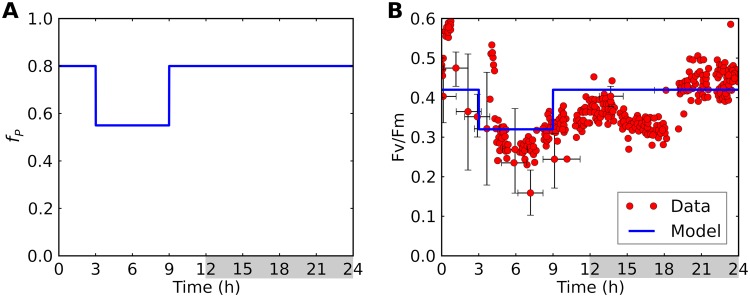
Modeled diurnal cycle of (A) the fraction of photosynthetic cells (*f_P_*) and (B) quantum yield (Fv/Fm). In panel B, model results (blue curves) are compared with observed data (red circles) (26). Light period, 0 to 12 h; dark period, 12 to 24 h (indicated by gray shading).

We run the model under a 12-h/12-h light/dark cycle, with *f_P_* prescribed as a step function in time (Fig. 3A). These transitions occur smoothly in nature and in experimental observations (26, 33, 34). Because our goal is to elucidate mechanisms, rather than to simulate precise details of particular experiments, we choose abrupt transitions that can be clearly discerned in the model output. As a check on the broad applicability of our diurnal forcing, we compared the trend of averaged Fv/Fm (photosynthetic quantum yield of photosystem II) to previous observations (26) (Fig. 3B). Fv/Fm is an indication on the efficiency of light use, which tends to be lower when cells are actively fixing nitrogen (26, 40). Also, it indicates the activity of photosystem II and thus the production of O_2_ (26, 41). The general trend in observations is captured by Fv/Fm = 0.5 and 0.1 for P cells and N cells, respectively. Similar values were observed during photosynthetic and nonphotosynthetic periods in *Crocosphaera* (40).

### Mechanisms of O_2_ management.

The maintenance of low intracellular O_2_ levels to permit N_2_ fixation can be achieved through several potential mechanisms, including the following: (i) trichome formation, (ii) respiratory protection, and (iii) diffusion barriers. All of these factors need to be included in order to reproduce the observed diurnal variation in metabolic rates (Fig. 1). Here we describe model results that evaluate the importance of each mechanism.

### Trichome formation.

**FIG 4:**
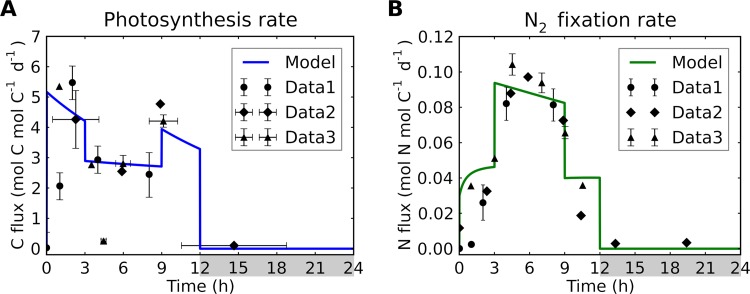
Rates of photosynthesis and N_2_ fixation for a simulated trichome. (A) C-based rates of photosynthesis. Data represent C fixation rates normalized from results reported in references 33 (Data1) and 26 (Data2 and Data3). Data3 results represent conversion from gross O_2_ evolution data. (B) N-based rates of nitrogen fixation. Data represent N_2_ fixation rates normalized from results reported in references 33 (Data1) and 26 (Data2 and Data3). For Data1, only daytime data are plotted. The normalized data are plotted using magnitudes similar to those of the model outputs. Model fluxes are normalized to biomass carbon levels. Light period, 0 to 12 h; dark period, 12 to 24 h (indicated by gray shading).

Due to the production of O_2_, N_2_ fixation cannot occur in the photosynthetic cells. Thus, trichome formation with differently functioning cells is a key factor for temporal coexistence of N_2_ fixation and photosynthesis. We reproduce the observed daily cycle of photosynthesis and N_2_ fixation (Fig. 4) by simulating the daily cycle of the fractions of P cells and N cells (Fig. 3A).

The observed cycle of photosynthesis with midmorning and midafternoon peaks (26, 33) (Fig. 1) is reproduced by the model. This is achieved by an increase in levels of N_2_-fixing cells (decreased *f_P_*) during the middle of the day (from h 3 to h 9). In the early morning, photosynthesis increases rapidly because of increased light levels, followed by a sharp decrease during the middle of the light period (Fig. 4A) due to decreasing levels of photosynthetic cells. After the colony shifts toward greater numbers of photosynthetic cells in the evening (h 9), the photosynthesis rate increases again but at lower rates than in early morning, a difference that is consistent with observations (Fig. 4A). In the model, this difference is due to C storage reaching the maximum capacity of finite cell volumes at the end of the light period. The levels of N cells increase at the expense of P cells, and the rate of N_2_ fixation rises. This temporal physiological shift has been experimentally observed when a decrease in the rate of photosynthesis coincided with an increase in the rate of N_2_ fixation (Fig. 4B) (26, 33). The model captures this trend with increased *f_N_* levels (and thus decreased *f_P_* levels), but does not do so if *f_N_* and *f_P_* levels are held constant (see Fig. S1 in the supplemental material), suggesting that diurnal shifts in metabolic function of cells are important.

10.1128/mSystems.00210-19.2 FIG S1 Rates of photosynthesis and N_2_ fixation for a simulated trichome with *f_P_* = 0.8 throughout the day. (A) C-based rates of photosynthesis. Data are C fixation rates normalized from references 1 (Data1) and 2 (Data2 and Data3). Data3 results represent conversion from gross O_2_ evolution data. (B) N-based rates of N_2_ fixation. Data are N_2_ fixation rates normalized from references 1 (Data1) and 2 (Data2 and Data3). For Data1, only daytime data are plotted. The normalized data are plotted using magnitudes similar to those of the model outputs. Model fluxes are normalized by biomass carbon. Light period, 0 to 12 h; dark period, 12 to 24 h (indicated by gray shading). Download FIG S1, PDF file, 0.3 MB. Copyright © 2019 Inomura et al. 2019 Inomura et al. This content is distributed under the terms of the Creative Commons Attribution 4.0 International license.

The rate of N_2_ fixation peaks during the middle of the day due to increased levels of nonphotosynthetic cells and accumulated C. During the period from ∼h 0 to ∼h 3, C stores accumulates due to high rates of photosynthesis, leading to a gradual increase in the rate of N_2_ fixation (Fig. 4B). When the trichomes shift toward greater numbers of N_2_-fixing cells (*f_P_* levels having decreased from 0.8 to 0.55) later in the morning (h 3), the rate of N_2_ fixation almost doubles. A smaller subsequent decrease is due to loss of C storage and simultaneously N storage getting closer to its maximum, both of which act to reduce *F*_Nfix_ rates (equation 1). In the evening (h 9), the rate is decreased by almost half due to the decreased presence of nonphotosynthetic cells before finally dropping to zero at the onset of the dark period.

### Respiratory protection.

**FIG 5:**
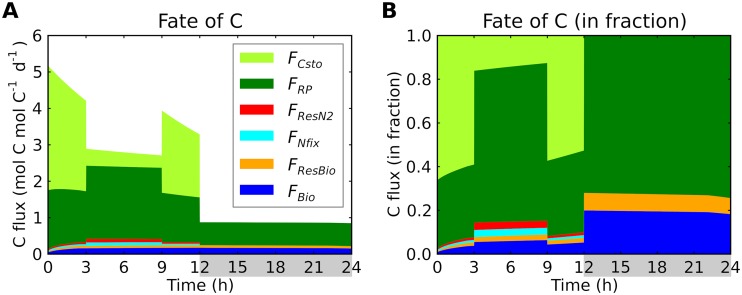
Diurnal allocation of C fluxes to modeled cellular functions. (A) C fluxes in moles of C per mole of C biomass per day. *F_Csto_*, C storage production; *F_RP_*, respiratory protection; *F_ResN2_*, respiration for providing energy for N_2_ fixation; *F_Nfix_*, carbon consumption for providing electrons for N_2_ fixation; *F_ResBio_* (=$F_{\mathrm{Res}}^{P})$, respiration for providing energy for biomass production; *F_Bio_*, biomass production. Light period, 0 to 12 h; dark period, 12 to 24 h (indicated by gray shading). During the light period, the origin of C is photosynthesis, while during the dark period, it is C storage. *F_ResN2_* is computed based on energetic balance (12, 45), and *F_RP_* represents the remaining respiration (see Text S1). (B) C fluxes in fraction.

Light harvesting is essential for providing the organic C and electrons needed to maintain low O_2_ concentrations via respiratory activity. Over 80% of C is used for respiratory protection during the light period, except for storage accumulation (Fig. 5). In comparison, the consumption of C to supply energy and electrons for N_2_ fixation was less than 10%. The fraction of C corresponding to respiratory protection was above 70% during the dark period (Fig. 5). This large quantity of C used for respiratory protection explains the previously observed differences between apparent levels of C fixation and O_2_ evolution. Under optimal conditions, the mean O_2_ production can reach rates of 26 mg O_2_ (mg Chl-a)^−1^ h^−1^, while the net C fixation rate is 4.5 mg C (mg Chl-a)^−1^ h^−1^ (10). The difference in respiration between day and night corresponds to light-dependent respiration levels; the predicted ratio of light-dependent respiration to dark respiration (Fig. 5) is close to what has been previously observed (42). The model also explains the unusually high basal rates of dark respiration observed in *Trichodesmium*: 0.18 μmol O_2_ (μg Chl-a)^−1^ h^−1^ (10, 42). Estimated rates of basal dark respiration for Skeletonema costatum and Pavlova lutheri are ∼5% to ∼6% of those for *Trichodesmium* (43, 44). This high respiration rate indicates that *Trichodesmium* maintains low intracellular O_2_ levels even during the dark period, possibly to maintain and/or synthesize nitrogenase.

The use of C/electron for respiratory protection comes at the expense of cellular growth and therefore may also explain the low growth rate of *Trichodesmium*. While non-nitrogen fixers have nutrient replete growth rate (μ_max_) of over 1 day^−1^ (45), that for *Trichodesmium* is about 0.1 to 0.5 day^−1^ (10, 45). Under conditions of nutrient repletion, the μ_max_ rate for the cell can be described as follows:

(2) $${\text{μ}}_{\text{max}}=F_{\text{Cfix}}^{Q_{c}}Y_{}^{\text{Bio:Cfix}}$$

where $F_{\text{Cfix}}^{Q_{c}}$ is the C fixation rate per cellular C quota (*Q_C_*) and *Y*_Bio:Cfix_ is the biomass yield of production for a given amount of C fixation. Because ∼80% of C is used for respiratory protection (Fig. 5), μ_max_ is reduced to ∼20% of its potential value, which is close to the observed difference in the μ_max_ values between *Trichodesmium* and other non-nitrogen-fixing phytoplankton (45). Thus, the energetic demands of maintaining the intracellular O_2_ at a level low enough for N_2_ fixation appears to explain the reduced growth rates of this species in relation to other phytoplankton. This growth rate handicap is a critical factor in plankton ecology (13, 14, 17) and in the dynamics of the global N cycle (19, 46). Without such a handicap, it is possible that *Trichodesmium* could outcompete non-nitrogen fixers even where N is not limited.

### Diffusion barriers.

**FIG 6:**
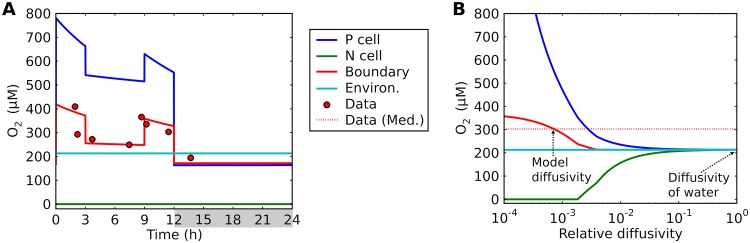
Model-data comparison of O_2_ concentrations. (A) Modeled time series of O_2_ with low diffusion coefficient based on the predicted low diffusivity; see “Model diffusivity” in panel B. (B) Modeled daytime average O_2_ concentrations for various levels of diffusivity of cell membrane layers relative to that of water at 25°C. The key in the center of the figure applies to both panel A and panel B. “P cell,” “N cell,” “Boundary,” and “Environ.” represent model results corresponding to boxes representing photosynthetic cells, nonphotosynthetic cells, the boundary layer, and the environment. Points in panel A represent time series data corresponding to the boundary layer (±300 μm from the center of the colony [34]); compare to “Boundary”. The horizontal dashed line in panel B indicates the median of the daytime data presented in panel A. Light period, 0 to 12 h; dark period, 12 to 24 h (indicated by gray shading in panel A).

Even in the absence of photosynthesis, diffusion of O_2_ from the ambient environment may result in O_2_ permeating the cell, disabling the active site of the nitrogenase enzyme. Published observations of O_2_ concentrations within the colony of *Trichodesmium* reveal strong diurnal variations in O_2_ concentrations in the boundary layer (Fig. 6A, red circles) (34), with maxima in the early morning and evening, echoing variations in photosynthesis rate. Although O_2_ is consistently supersaturated in the boundary layer during the day, its levels become slightly lower during the night. These observations provide a key constraint on the model representation of O_2_ diffusion and the importance of the *Trichodesmium* strategy to protect N_2_ fixation by minimizing diffusive O_2_ fluxes (Fig. 6).

The observed trends in levels of O_2_ in the boundary layer can be captured when we apply low diffusivity for cell membrane layers (lower than 10^−3^ of that in water) (here referred to as “model diffusivity”) (Fig. 6A and B). First, we predict consistently higher levels of O_2_ in the boundary layer than in the environment, as observed (34) (Fig. 6A). Despite the respiratory protection, net O_2_ production rates are still positive, increasing the levels of boundary layer O_2_. Second, [O_2_]*_B_* reached two peaks: each peak during the early and later parts of the day. This high O_2_ concentration is due to high rates of photosynthesis (Fig. 4A). The peak value during early light period is slightly higher (∼400 μM) than that in the evening, consistent with the observations (34). The O_2_ levels decrease during the middle of the day, due to the decreased fraction of photosynthetic cells. However, the concentration (∼300 μM) is still higher than the O_2_ concentration in the environment [O_2_]*_E_*, also consistent with the observations. The higher value occurs based on the balance between respiration rates and photosynthesis; if the number of photosynthetic cells decreases, [O_2_]*_B_* can become lower than [O_2_]*_E_*. During the dark period, since there is no photosynthesis, the model predicts lower [O_2_]*_B_* (< 200 μM) than [O_2_]*_E_*, as previously observed (34), due to respiration.

Intracellular and boundary layer O_2_ concentrations are highly sensitive to the levels of diffusivity (Fig. 6B), supporting the importance of strong diffusion barriers. Decreasing diffusivity would increase the passive uptake of O_2_ by N cells, requiring a higher amount of C. For diffusivity levels exceeding twice that of our default model diffusivity, intracellular O_2_ cannot be maintained at the minimum level. Also, when the diffusivity is higher than three times the default value, the boundary layer O_2_ concentration becomes similar to that in the environment, failing to reproduce higher concentrations in the boundary layer environment. If we assume diffusivity of water for the cellular membrane (47), all the boxes have similar averaged O_2_ levels (Fig. 6B), and N_2_ fixation cannot be maintained. The predicted low diffusivity is qualitatively consistent with the results of a recent study (38).

## DISCUSSION

### Potential explanations for low diffusivity.

One explanation for the low level of model diffusivity is the low diffusivity of the bacterial membranes. *Trichodesmium* is a Gram-negative bacterium whose cell envelope has multiple layers (48), with inner and outer lipid membranes separated by a periplasm containing peptidoglycan. The outer lipid membrane is connected to lipopolysaccharide (LPS), creating a capsule. Possibly due to the presence of these layers, the O_2_ diffusivity of bacterial membranes is predicted to be ∼2 to ∼3 orders of magnitude lower than that of water (12, 49, 50). The diffusivity of cells with simple lipid bilayers is generally within the same order of magnitude as that of water (50). Thus, it is likely that the layers of LPS or peptidoglycan provide strong barriers against O_2_ in *Trichodesmium*.

Although the Gram-negative membrane might be sufficient, another potential mechanism for the diffusion barrier may be production of extracellular polymeric substances (EPS). Recent studies suggest involvement of EPS in protecting heterotrophic nitrogen fixers of the species Azotobacter vinelandii (12, 51, 52). This has been supported by a laboratory study where respiration decreases with EPS production (53). *Trichodesmium* produces EPS especially under nutrient-limited conditions (54–56). Here, given the low model diffusivity, another purpose of EPS might be the management of O_2_ from the environment, which accounts for over 80% of O_2_ input in model simulations (see Fig. S2 in the supplemental material). The remaining ∼10% to ∼20% of O_2_ input is directly transported from neighboring photosynthetic cells, and this intercellular flux must also be minimized.

10.1128/mSystems.00210-19.3FIG S2Two different O_2_ fluxes into nonphotosynthetic cells. $J_{O2}^{\mathrm{PN}}$, O_2_ flux directly from the photosynthetic cells. $J_{O2}^{\mathrm{BN}}$, O_2_ flux from the boundary layer. Light period, 0 to 12 h; dark period, 12 to 24 h (indicated by gray shading). Download FIG S2, TIF file, 0.3 MB.Copyright © 2019 Inomura et al.2019Inomura et al.This content is distributed under the terms of the Creative Commons Attribution 4.0 International license.

To reproduce the observed fluctuations of O_2_ concentration in the boundary layer, the model required 45% of nonphotosynthetic cells during the middle of the light period; fractions below 45% result in insufficient diel fluctuations of O_2_. This value sits between two observational results; some studies show almost all the cells having nitrogenase (30, 57), whereas other laboratory studies show that only ∼10% to ∼20% of cells actually contain nitrogenase (24, 31, 32). In the latter case, nitrogenase may be confined to a subset of nonphotosynthetic cells.

**FIG 7:**
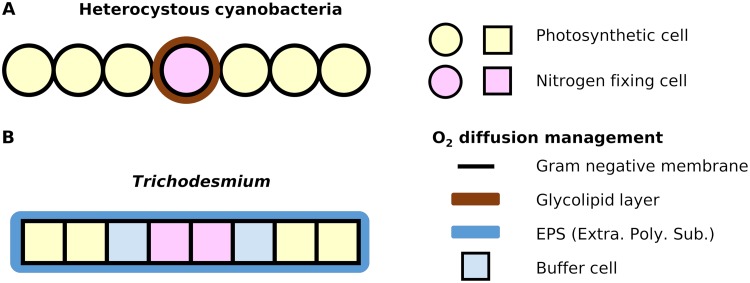
Model-based hypothesis of how *Trichodesmium* manages O_2_ during the light period in comparison to heterocystous cyanobacteria. (A) General understanding of how heterocystous cyanobacteria manage O_2_. (B) Proposed O_2_ management by *Trichodesmium*. The key at the right side of the figure applies to both panel A and panel B. “EPS” represents extracellular polymeric substances (Extra. Poly. Sub.). The model predicts that N_2_-fixing cells and buffer cells in *Trichodesmium* would have high respiration for O_2_ scavenging (respiratory protection). The Gram-negative membranes between cells are doubled. The locations of these cells may be switching in the time scale of minutes, but an anoxic environment can be created instantly (i.e., within seconds) after such a switch due to the high rate of respiration relative to the intracellular O_2_ concentration.

Given the predicted low diffusivity, certain cells may play a role in reducing O_2_ diffusion from photosynthetic cells to N_2_-fixing cells. On the basis of this hypothesized function, we refer to them as “buffer cells.” Because cell membranes have significantly slower diffusion and higher viscosity than water, having more membranes between P cells and N cells containing nitrogenase would decrease O_2_ transport between them. Because EPS can prevent O_2_ diffusion from the environment only, such buffer cells may represent an alternative to the glycolipid layers seen in heterocysts and thus may be essential for the coexistence of photosynthesis and N_2_ fixation (Fig. 7).

The double layer of Gram-negative membranes between two adjacent cells could effectively reduce O_2_ transport. However, such cells and membranes could also slow the transport of carbon or other reduced molecules. To circumvent such problems, we hypothesize that cells may selectively transfer molecules. Such a mechanism seems to exist in heterocystous cyanobacteria, which rapidly transfer sucrose through cell junctions (58). It is also possible that sites for N_2_ fixation occur where C storage is sufficiently accumulated. This would explain why the lowest fractions of N_2_-fixing cells are observed just before dawn, when cellular C storage is most depleted (24, 31).

### The residence time of O_2_ is significantly shorter than time scales of metabolic switching.

Fluorescence kinetics data show various states of photosynthetic activity throughout a trichome (41). However, until now, it has not been resolved whether cellular O_2_ management strategies can keep up with changes in photosynthetic activity, which occur over time scales of minutes (25). Since our model focuses on the averaged state of a trichome based on the fraction of different metabolisms (photosynthetic or nitrogen fixing), it does not resolve such dynamic locational shifts of photosynthetic activity. It does show, however, that the residence time of O_2_ is extremely short, on the order of 1 s. The predicted daytime rate of respiration is ∼2 mol O_2_ mol C^−1^ day^−1^, equivalent to 0.424 mol O_2_ m^−3^ s^−1^ with a cellular C concentration of 1.83 × 10^4^ mol m^−3^ (59). For a typical concentration of O_2_ in tropical surface water (∼0.2 mol m^−3^), the time scale of O_2_ turnover is ∼0.5 s. Even if we apply our predicted O_2_ concentration in photosynthetic cells (∼0.6 mol m^−3^), the time scale is only ∼1.4 s. This simple calculation indicates the potential for photosynthetic cells to quickly switch to nitrogen-fixing cells as long as nitrogenase can be preserved within the photosynthetic cells. The capability of preserving nitrogenase may be supported by evidence indicating that nitrogenase is found in most of the cells (30, 57). However, how nitrogenase is preserved during photosynthesis needs to be further investigated. Potential mechanisms include “conformational protection,” in which the activity of nitrogenase is rapidly and reversibly switched off in response to high O_2_ concentrations (2, 60, 61), thereby limiting the time-consuming processes of resynthesizing or repairing nitrogenases.

### Broader context: how can photosynthesis and N_2_ fixation occur simultaneously in general?

Nitrogenase is highly sensitive to O_2_ (5, 6, 9); therefore, photosynthetic O_2_ production is detrimental to N_2_ fixation. However, some major nitrogen fixers and their symbioses enable these conflicting processes to occur simultaneously; e.g., *Trichodesmium*, diatom diazotroph association (DDA) (62, 63), and UCYN-A and phytoplankton symbiosis (64). Here, on the basis of a simple model of *Trichodesmium*, we discuss how these processes can coexist over short distances.

First, N_2_ fixation and photosynthesis should not occur in the same cell simultaneously. Nitrogenase exists in the cytoplasm, while photosynthesis occurs on the intracytoplasmic membranes. Thus, photosynthesis would likely release O_2_ into cytoplasm, damaging nitrogenase. Also, we predict that rates of respiration exceeding that of photosynthesis would be required for consuming this O_2_. This would not be sustainable in cells that lack metabolic specialization. On the other hand, if these processes occur in different cells, the amount of O_2_ that needs to be managed decreases significantly. Thus, it would make sense that such functional separation has been selected through evolution.

Second, diffusivity control of O_2_ and high respiration rates must occur together, unless diffusivity is perfectly controlled. The model uses low diffusivity through membranes, but it still requires high respiration rates. High respiration has been reported not only in *Trichodesmium*, but also *Crocosphaera*, unicellular photoautotrophic nitrogen fixers (11). Organisms with heterocysts may not require such high levels of respiration with sophisticated O_2_ barriers. It is likely that decreasing the diffusion can affect the uptake/transfer of other important nutrients/metabolites. Thus, for such strong barriers, selective transfer of organic C substrates to fuel respiration must be involved.

### Conclusions.

With a mechanistic model of *Trichodesmium*, we investigate how these photosynthetic cyanobacteria manage to fix nitrogen while also fixing carbon. The model reproduces observed patterns of diel cycling in *Trichodesmium* physiology and indicates that these conflicting processes must occur in different cells and also need to be separated by a barrier to O_2_ diffusion. These results support the hypothesis that a Gram-negative membrane could represent an essential diffusion barrier against O_2_. EPS and buffer cells might additionally decrease O_2_ diffusion. The remaining O_2_ must be consumed through respiratory protection for N_2_ fixation to occur, and such respiration may explain the observed low growth rate of *Trichodesmium*. The model indicates that the residence time of O_2_ within trichomes is a few seconds, significantly shorter than the time scale of switching on and off photosynthesis. Our model and the results provide a theoretical basis for investigating how these two conflicting processes occur in one of the major sources of new N to the oligotrophic surface ocean.

## MATERIALS AND METHODS

### Model equations.

In this section, we explain the assumptions and equations used in the model. This model resolves C, N, and O_2_ budgets (Fig. 2) within cells, as well as the O_2_ budget in the immediate vicinity (boundary layer) of those cells. The budgets consist of biochemical rates within each type of cell and exchange between cells of each type and between cells and the environment. At any given time, these rates depend on the fraction of the trichome that is assigned to each metabolic function. We denote *f*_P_ and *f*_N_ as the fractions of photosynthetic and nonphotosynthetic cells, respectively. Of the nonphotosynthetic cells (*f*_N_), a constant fraction (*f*_NITROGE_) is assumed to contain nitrogenase and thus to be able to fix N_2_. The detailed nomenclature (with units) is provided in Table S1 in the supplemental material. To determine the rate of N_2_ fixation (equation 1), we need to specify N and C storage and cellular O_2_ in the N_2_-fixing cells. Since these quantities are themselves governed by an allocation of resources that varies with environmental conditions, we model these variables with a set of differential equations. The difference between C production (photosynthesis) and consumption (respiration, N_2_ fixation, and biomass production) leads to the change in C storage (*C*_Sto_). Similarly, the change in N storage (*N*_Sto_) is governed by the balance of N_2_ fixation and biomass production as represented in the following equations:

(3) $$\frac{dC_{\text{Sto}}}{\mathrm{dt}}=F_{\text{Cfix}}^{}-F_{\text{Bio}}^{}-F_{\text{Nfix}}^{}Y_{\text{Nfix}}^{C\text{:}N}-F_{\text{Res}}^{}$$

(4) $$\frac{dN_{\text{Sto}}}{\mathrm{dt}}=F_{\text{Nfix}}-F_{\text{Bio}}^{}Y_{\text{Bio}}^{N\text{:}C}$$

where *C*_Sto_ represents C storage, *t* represents time, *F*_Cfix_ represents C fixation rate, *F*_Bio_ represents biomass production rate, *F*_Nfix_ represents N_2_ fixation rate, $Y_{\text{Nfix}}^{C\text{:}N}$ represents a C:N conversion term for N_2_ fixation, *F*_Res_ represents respiration rate, *N*_Sto_ represents N storage, and $Y_{\text{Bio}}^{N\text{:}C}$ represents N:C of biomass.

10.1128/mSystems.00210-19.4 TABLE S1 A list of the parameters roughly in order of appearance in the main text and Text S1. Download Table S1, PDF file, 0.2 MB. Copyright © 2019 Inomura et al. 2019 Inomura et al. This content is distributed under the terms of the Creative Commons Attribution 4.0 International license.

*C*_Sto_ and *N*_Sto_ are governed by 2 common rates (*F*_Nfix_ and *F*_Bio_), whereas *C*_Sto_ has additional input from C fixation and respiration that need to be modeled. The *C*_Sto_ and *N*_Sto_ values are computed with a finite-difference method and therefore cause temporal variations in C:N values.

We assume that the rate of biomass production (*F*_Bio_) is a minimum function of available storage resources (*C*_Sto_ and *N*_Sto_), calculated as follows:

(5) $$F_{\text{Bio}}^{}=F_{\text{Bio}}^{\text{max}}\text{ min}\left(\frac{C_{\text{Sto}}}{C_{\text{Sto}}+K_{C}},\;\frac{N_{\text{Sto}}}{N_{\text{Sto}}+K_{N}}\right)$$

where $K_{N}=K_{C}Y_{\text{B}}{{}_{\text{io}}}^{\left(N:C\right)}$.

The rate of C fixation (*F*_Cfix_) is proportional to the level of chlorophyll available for photosynthesis:

(6) $$F_{\text{Cfix}}^{}=F_{\text{Cfix}}^{\text{Chl}}{\text{Chl}}_{P}$$

where

(7) $$F_{\text{Cfix}}^{\text{Chl}}=F_{\text{CfixMax}}^{\text{Chl}}\left(1-e_{}^{-K_{I}I}\right)\left(C_{\text{Sto}}^{\text{max}}-C_{\text{Sto}}\right)$$

Here C fixation saturates with light and slows as C storage approaches its maximum capacity. We assume that the level of chlorophyll per P cell is constant and proportional to the fraction of P cells *f_P_* (= 1 – *f_N_*) since the model runs at constant light during the light period as follows:

(8) $${\text{Chl}}_{P}=f_{P}^{}{\text{Chl}}_{\text{full}}^{}$$

where Chl_full_ is chlorophyll content when all the cells are carbon fixing; thus Chl*_C_* = Chl_full_ when *f_P_* = 1.

The total respiration rate (*F*_Res_) is the sum of the rates in P cells ($F_{\text{Res}}^{P}$) and in N cells ($F_{\text{Res}}^{N}$) and is calculated as follows:

(9) $$F_{\text{Res}}^{}=F_{\text{Res}}^{P}+F_{\text{Res}}^{N}$$

We assume that biosynthesis occurs mostly in P cells, and the respiration for supporting the biosynthesis assumed to be proportional with a constant ratio of *Y*_Res:Bio_, leading to the following equation:

(10) $$F_{\text{Res}}^{P}=F_{\text{Bio}}^{}Y_{}^{\text{Res:Bio}}$$

To compute $F_{\text{Res}}^{N}$, we use the O_2_ balances as follows:

(11) $$\frac{V_{P}}{V}\frac{d{\left[{\text{O}}_{2}\right]}_{P}}{\mathrm{dt}}=-J_{{\text{O}}_{2}}^{\mathrm{PB}}-J_{{\text{O}}_{2}}^{\mathrm{PN}}+\left(F_{\text{Cfix}}^{}-F_{\text{Res}}^{P}\right)Y_{}^{{\text{O}}_{2}:C}$$

(12) $$\frac{V_{N}}{V}\frac{d{\left[{\text{O}}_{2}\right]}_{N}}{\mathrm{dt}}=J_{{\text{O}}_{2}}^{\mathrm{PN}}+J_{{\text{O}}_{2}}^{\mathrm{BN}}-F_{\text{Res}}^{N}Y_{}^{{\text{O}}_{2}:C}$$

(13) $$\frac{V_{B}}{V}\frac{d{\left[{\text{O}}_{2}\right]}_{B}}{\mathrm{dt}}=-J_{{\text{O}}_{2}}^{\mathrm{BE}}+J_{{\text{O}}_{2}}^{\mathrm{PB}}-J_{{\text{O}}_{2}}^{\mathrm{BN}}$$

Here, *V_i_* represents the volume of box *i* (*i *=* P*, *N*, or *B*, representing boxes for photosynthetic cells, nonphotosynthetic cells, or boundary layer, respectively); *V* = *V_P_*+*V_N_*, [O_2_]_*i*_ represents the O_2_ concentration of box *i* (*i *=* P*, *N*, or *B*); $J_{{\text{O}}_{2}}^{\mathrm{ij}}$ represents O_2_ diffusion from box *i* to *j* (*i*, *j *=* P*, *N*, *B*, or *E*, where *E* represents a box for the environment); $J_{{\text{O}}_{2}}^{\mathrm{ij}}=A_{\mathrm{ij}}\left({\left[{\text{O}}_{2}\right]}_{i}-{\left[{\text{O}}_{2}\right]}_{j}\right)$ represents *A_ij_*, the diffusion coefficient from box *i* to *j* (*i*, *j *=* P*, *N*, *B*, or *E*); $F_{\text{Res}}^{i}$ represents the respiration rate in box *i* (*i= P* or *N*) ($F_{\text{Res}}^{P}$ + $F_{\text{Res}}^{N}$ = *F_Res_*); and *Y*^O_2_:*C*^ represents C-to-O_2_ conversion in respiration and C fixation.

Since the time scale of O_2_ equilibration is much smaller than that of N and C, we assume a pseudo-steady state of O_2_. This assumption largely applies since the cellular concentrations of C and N are on the order of 10^4^ and 10^3^ (mol m^−3^), respectively, while that of O_2_ is generally below 1 (mol m^−3^) despite the fact that the magnitudes of the fluxes are similar. The pseudo-steady-state assumption gives a single solution for $F_{\text{Res}}^{N}$ as well as for the O_2_ concentration in each box. Since $F_{\text{Res}}^{N}$ needs to be supported by carbon, in cases where carbon availability becomes limited, $F_{\text{Res}}^{N}$ becomes limited as well, in which case the O_2_ concentration of N_2_-fixing cells increases and limits the rate of N_2_ fixation (equation 1). Detailed explanations of O_2_ balance and computation of $F_{\text{Res}}^{N}$ and O_2_ concentrations are provided in Text S1 in the supplemental material.

### Parameterization.

The model requires specification of several parameters which are obtained from previous studies (Table S2). These include elemental stoichiometry of biomass and boundary conditions such as light intensities and O_2_ concentrations in the environment. The remaining parameters are selected to reproduce the observed metabolic rates of *Trichodesmium* as compiled from multiple published studies (10, 27, 29, 42, 65–67) (Table S3). In general, the key metabolic processes follow a well-defined order, with N_2_ fixation being the slowest (∼0.006 to ∼0.146 mol N mol biomass C^−1^ day^−1^), O_2_ production being the fastest (∼1.10 to ∼288 mol O_2_ mol biomass C^−1^ day^−1^), and net C fixation being intermediate between the other two (∼0.16 to ∼2.57 mol C mol biomass C^−1^ day^−1^). The remaining parameters have been tuned to bring averaged model output values within these ranges.

10.1128/mSystems.00210-19.5 TABLE S2 A list of the values used for fixed parameters. Download Table S2, PDF file, 0.2 MB. Copyright © 2019 Inomura et al. 2019 Inomura et al. This content is distributed under the terms of the Creative Commons Attribution 4.0 International license.

10.1128/mSystems.00210-19.6 TABLE S3 A list of the values used for adjustable parameters. Download Table S3, PDF file, 0.1 MB. Copyright © 2019 Inomura et al. 2019 Inomura et al. This content is distributed under the terms of the Creative Commons Attribution 4.0 International license.

### Model availability.

The model in this study was written in Python 3 and is freely available at https://zenodo.org/record/1245128.

## References

[1] Karl D, Letelier R, Tupas L, Dore J, Christian J, Hebel D 1997 The role of nitrogen fixation in biogeochemical cycling in the subtropical North Pacific Ocean. Nature 388:533–538. doi:10.1038/41474.

[2] Robson RL, Postgate JR 1980 Oxygen and hydrogen in biological nitrogen fixation. Annu Rev Microbiol 34:183–207. doi:10.1146/annurev.mi.34.100180.001151. 6776883

[3] Postgate JR 1982 Biological nitrogen fixation: fundamentals. Philos Trans R Soc B 296:375–385. doi:10.1098/rstb.1982.0013.

[4] Sohm JA, Webb EA, Capone DG 2011 Emerging patterns of marine nitrogen fixation. Nat Rev Microbiol 9:499–508. doi:10.1038/nrmicro2594. 21677685

[5] Gallon JR 1981 The oxygen sensitivity of nitrogenase: a problem for biochemists and micro-organisms. Trends Biochem Sci 6:19–23. doi:10.1016/0968-0004(81)90008-6.

[6] Wang ZC, Burns A, Watt GD 1985 Complex formation and O_2_ sensitivity of *Azotobacter vinelandii* nitrogenase and its component proteins. Biochemistry 24:214–221. doi:10.1021/bi00322a031. 2986674

[7] Berman-Frank I, Chen Y-B, Gao Y, Fennel K, Follows MJ, Milligan AJ, Falkowski P 2008 Feedbacks between the nitrogen, carbon and oxygen cycles, p 1537–1563. *In* Capone DG, Bronk DA, Mulholland MR, Carpenter EJ, Nitrogen in the marine environment, 2nd ed Elsevier, Burlington, VT.

[8] Maldener I, Muro-Pastor AM 2010 Cyanobacterial heterocysts *In* Encyclopedia of life sciences (eLS). John Wiley & Sons, Ltd, Chichester, United Kingdom.

[9] Poole RK, Hill S 1997 Respiratory protection of nitrogenase activity in *Azotobacter vinelandii*: roles of the terminal oxidases. Biosci Rep 17:303–317. doi:10.1023/a:1027336712748. 9337485

[10] LaRoche J, Breitbarth E 2005 Importance of the diazotrophs as a source of new nitrogen in the ocean. J Sea Res 53:67–91. doi:10.1016/j.seares.2004.05.005.

[11] Großkopf T, LaRoche J 2012 Direct and indirect costs of dinitrogen fixation in *Crocosphaera watsonii* WH8501 and possible implications for the nitrogen cycle. Front Microbiol 3 doi:10.3389/fmicb.2012.00236. PMC3401090 22833737

[12] Inomura K, Bragg J, Follows MJ 2017 A quantitative analysis of the direct and indirect costs of nitrogen fixation: a model based on *Azotobacter vinelandii*. ISME J 11:166–175. doi:10.1038/ismej.2016.97. 27740611 PMC5315487

[13] Barton AD, Pershing AJ, Litchman E, Record NR, Edwards KF, Finkel ZV, Kiørboe T, Ward BA 2013 The biogeography of marine plankton traits. Ecol Lett 16:522–534. doi:10.1111/ele.12063. 23360597

[14] Monteiro FM, Follows MJ, Dutkiewicz S 2010 Distribution of diverse nitrogen fixers in the global ocean. Global Biogeochem Cycles 24:GB3017. doi:10.1029/2009GB003731.

[15] Monteiro FM, Dutkiewicz S, Follows MJ 2011 Biogeographical controls on the marine nitrogen fixers. Global Biogeochem Cycles 25:GB2003. doi:10.1029/2010GB003902.

[16] Yoshikawa C, Coles VJ, Hood RR, Capone DG, Yoshida N 2013 Modeling how surface nitrogen ﬁxation inﬂuences subsurface nutrient patterns in the North Atlantic. J Geophys Res Oceans 118:2520–2534. doi:10.1002/jgrc.20165.

[17] Stukel MR, Coles VJ, Brooks MT, Hood RR 2014 Top-down, bottom-up and physical controls on diatom-diazotroph assemblage growth in the Amazon River plume. Biogeosciences 11:3259–3278. doi:10.5194/bg-11-3259-2014.

[18] Tyrrell T 1999 The relative influences of nitrogen and phosphorus on oceanic primary production. Nature 400:525–531. doi:10.1038/22941.

[19] Weber TS, Deutsch C 2012 Oceanic nitrogen reservoir regulated by plankton diversity and ocean circulation. Nature 489:419–422. doi:10.1038/nature11357. 22996557

[20] Capone DG, Zehr JP, Paerl HW, Bergman B, Carpenter EJ 1997 *Trichodesmium*, a globally significant marine cyanobacterium. Science 276:1221–1229. doi:10.1126/science.276.5316.1221.

[21] Hood RR 2000 Answers sought to the enigma of marine nitrogen fixation. EOS 81:138–139.

[22] Capone DG, Carpenter EJ 1982 Nitrogen fixation in marine environment. Science 217:1140–1142. doi:10.1126/science.217.4565.1140. 17740970

[23] Subramaniam A, Brown CW, Hood RR, Carpenter EJ, Capone DG 2001 Detecting *Trichodesmium* blooms in SeaWiFS imagery. Deep Res II 49:107–121. doi:10.1016/S0967-0645(01)00096-0.

[24] El-Shehawy R, Lugomela C, Ernst A, Bergman B 2003 Diurnal expression of *hetR* and diazocyte development in the filamentous non-heterocystous cyanobacterium *Trichodesmium erythraeum*. Microbiology 149:1139–1146. doi:10.1099/mic.0.26170-0. 12724375

[25] Zehr JP 2011 Nitrogen fixation by marine cyanobacteria. Trends Microbiol 19:162–173. doi:10.1016/j.tim.2010.12.004. 21227699

[26] Berman-Frank I, Lundgren P, Chen Y-B, Küpper H, Kolber Z, Bergman B, Falkowski P 2001 Segregation of nitrogen fixation and oxygenic photosynthesis in the marine cyanobacterium *Trichodesmium*. Science 294:1534–1537. doi:10.1126/science.1064082. 11711677

[27] Wilson ST, Kolber ZS, Tozzi S, Zehr JP, Karl DM 3 5 2012, posting date. Nitrogen fixation, hydrogen cycling, and electron transport kinetics in *Trichodesmium erythraeum* (cyanobacteria) strain IMS101. J Phycol doi:10.1111/j.1529-8817.2012.01166.x. 27011075

[28] Paerl HW, Bebout BM 1988 Direct measurement of O_2_-depleted microzones in marine Oscillatoria: relation to N_2_ fixation. Science 241:442–445. doi:10.1126/science.241.4864.442. 17792609

[29] Paerl HW 1994 Spatial segregation of CO_2_ fixation in *Trichodesmium* spp.: linkage to N_2_ fixation potential. J Phycol 30:790–799. doi:10.1111/j.0022-3646.1994.00790.x.

[30] Ohki K 2008 Intercellular localization of nitrogenase in a non-heterocystous cyanobacterium (cyanophyte), *Trichodesmium* sp. NIBB1067. J Oceanogr 64:211–216. doi:10.1007/s10872-008-0016-2.

[31] Fredriksson C, Bergman B 1995 Nitrogenase quantity varies diurnally in a subset of cells within colonies of the non-heterocystous cyanobacteria *Trichodesmium*. Microbiology 141:2471–2478. doi:10.1099/13500872-141-10-2471.

[32] Lin S, Henze S, Lundgren P, Bergman B, Carpenter EJ 1998 Whole-cell immunolocalization of nitrogenase in marine diazotrophic cyanobacteria, *Trichodesmium* spp. Appl Environ Microbiol 64:3052–3058. 9687472 10.1128/aem.64.8.3052-3058.1998 PMC106814

[33] Finzi-Hart JA, Pett-Ridge J, Weber PK, Popa R, Fallon SJ, Gunderson T, Hutcheon ID, Nealson KH, Capone DG 2009 Fixation and fate of C and N in the cyanobacterium *Trichodesmium* using nanometer-scale secondary ion mass spectrometry. Proc Natl Acad Sci 106:6345–6350. doi:10.1073/pnas.0810547106. 19332780 PMC2669351

[34] Eichner MJ, Klawonn I, Wilson ST, Littmann S, Whitehouse MJ, Church MJ, Kuypers MMM, Karl DM, Ploug H 2017 Chemical microenvironments and single-cell carbon and nitrogen uptake in field-collected colonies of *Trichodesmium* under different *p*CO_2_. ISME J 11:1305–1317. doi:10.1038/ismej.2017.15. 28398346 PMC5437350

[35] Carpenter EJ, Price CC 1977 Nitrogen fixation, distribution, and production of Oscillatoria (*Trichodesmium*) spp. in the western Sargasso and Caribbean Seas. Limnol Oceanogr 22:60–72. doi:10.4319/lo.1977.22.1.0060.

[36] Carpenter E, Chang J, Cottrell M, Schubauer J, Paerl H, Bebout B, Capone D 1990 Re-evaluation of nitrogenase oxygen-protective mechanisms in the planktonic marine cyanobacterium *Trichodesmium*. Mar Ecol Prog Ser 65:151–158. doi:10.3354/meps065151.

[37] White AE, Watkins-Brandt KS, Church MJ 2018 Temporal variability of *Trichodesmium* spp. and diatom-diazotroph assemblages in the North Pacific subtropical gyre. Front Mar Sci 5:1–12.29552559

[38] Eichner M, Thoms S, Rost B, Mohr W, Ahmerkamp S, Ploug H, Kuypers MMM, Beer D 2019 N_2_ fixation in free-floating filaments of *Trichodesmium* is higher than in transiently suboxic colony microenvironments. New Phytol 222:852–863. doi:10.1111/nph.15621. 30507001 PMC6590460

[39] Rabouille S, Staal M, Stal LJ, Soetaert K 2006 Modeling the dynamic regulation of nitrogen fixation in the cyanobacterium *Trichodesmium* sp. Appl Environ Microbiol 72:3217–3227. doi:10.1128/AEM.72.5.3217-3227.2006. 16672460 PMC1472389

[40] Wilson ST, Tozzi S, Foster RA, Ilikchyan I, Kolber ZS, Zehr JP, Karl DM 2010 Hydrogen cycling by the unicellular marine diazotroph *Crocosphaera watsonii* strain WH8501. Appl Environ Microbiol 76:6797–6803. doi:10.1128/AEM.01202-10. 20709832 PMC2953037

[41] Küpper H, Ferimazova N, Šetlík I, Berman-Frank I 2004 Traffic lights in *Trichodesmium*. Regulation of photosynthesis for nitrogen fixation studied by chlorophyll fluorescence kinetic microscopy. Plant Physiol 135:2120–2133. doi:10.1104/pp.104.045963. 15299119 PMC520784

[42] Kana TM 1993 Rapid oxygen cycling in *Trichodesmium thiebautii*. Limnol Oceanogr 38:18–24. doi:10.4319/lo.1993.38.1.0018.

[43] Sakshaug E, Andresen K, Kiefer DA 1989 A steady state description of growth and light absorption in the marine planktonic diatom *Skeletonema costatum*. Limnol Oceanogr 34:198–205. doi:10.4319/lo.1989.34.1.0198.

[44] Chalup MS, Laws EA 1990 A test of the assumptions and predictions of recent microalgal growth models with the marine phytoplankter *Pavlova lutheri*. Limnol Oceanogr 35:583–596. doi:10.4319/lo.1990.35.3.0583.

[45] Follett CL, Dutkiewicz S, Karl DM, Inomura K, Follows MJ 2018 Seasonal resource conditions favor a summertime increase in North Pacific diatom–diazotroph associations. ISME J 12:1543. doi:10.1038/s41396-017-0012-x. 29449611 PMC5955908

[46] Weber T, Deutsch C 2014 Local versus basin-scale limitation of marine nitrogen fixation. Proc Natl Acad Sci U S A 111:8741–8746. doi:10.1073/pnas.1317193111. 24889607 PMC4066478

[47] Staal M, Meysman FJR, Stal LJ 2003 Temperature excludes N_2_-fixing heterocystous cyanobacteria in the tropical oceans. Nature 425:504–507. doi:10.1038/nature01999. 14523445

[48] Miller SI, Salama NR 2018 The gram-negative bacterial periplasm: size matters. PLoS Biol 16:e2004935. doi:10.1371/journal.pbio.2004935. 29342145 PMC5771553

[49] Inomura K, Bragg J, Riemann L, Follows MJ 2018 A quantitative model of nitrogen fixation in the presence of ammonium. PLoS One 13:e0208282. doi:10.1371/journal.pone.0208282. 30496286 PMC6264846

[50] MacDougall JDB, McCabe M 1967 Diffusion coefficient of oxygen through tissues. Nature 215:1173–1174. doi:10.1038/2151173a0. 6061810

[51] Sabra W, Zeng AP, Lünsdorf H, Deckwer WD 2000 Effect of oxygen on formation and structure of *Azotobacter vinelandii* alginate and its role in protecting nitrogenase. Appl Environ Microbiol 66:4037–4044. doi:10.1128/aem.66.9.4037-4044.2000. 10966426 PMC92256

[52] Wang D, Xu A, Elmerich C, Ma LZ 24 3 2017, posting date. Biofilm formation enables free-living nitrogen-fixing rhizobacteria to fix nitrogen under aerobic conditions. ISME J doi:10.1038/ismej.2017.30. PMC5520150 28338674

[53] Castillo T, López I, Flores C, Segura D, García A, Galindo E, Peña C 2018 Oxygen uptake rate in alginate producer (*algU*+) and nonproducer (*algU*-) strains of *Azotobacter vinelandii* under nitrogen-fixation conditions. J Appl Microbiol 125:181–189. doi:10.1111/jam.13760. 29573518

[54] Spungin D, Pfreundt U, Berthelot H, Bonnet S, AlRoumi D, Natale F, Hess WR, Bidle KD, Berman-Frank I 2016 Mechanisms of *Trichodesmium* demise within the New Caledonian lagoon during the VAHINE mesocosm experiment. Biogeosciences 13:4187–4203. doi:10.5194/bg-13-4187-2016.

[55] Bar-Zeev E, Avishay I, Bidle KD, Berman-Frank I 2013 Programmed cell death in the marine cyanobacterium *Trichodesmium* mediates carbon and nitrogen export. ISME J 7:2340–2348. doi:10.1038/ismej.2013.121. 23887173 PMC3834853

[56] Berman-Frank I, Rosenberg G, Levitan O, Haramaty L, Mari X 2007 Coupling between autocatalytic cell death and transparent exopolymeric particle production in the marine cyanobacterium *Trichodesmium*. Environ Microbiol 9:1415–1422. doi:10.1111/j.1462-2920.2007.01257.x. 17504479

[57] Paerl HW, Priscu JC, Brawner DL 1989 Immunochemical localization of nitrogenase in marine Trichodesmium aggregates: relationship to N(2) fixation potential. Appl Environ Microbiol 55:2965–2975. 16348057 10.1128/aem.55.11.2965-2975.1989 PMC203199

[58] Nürnberg DJ, Mariscal V, Bornikoel J, Nieves-Morión M, Krauß N, Herrero A, Maldener I, Flores E, Mullineaux W 2015 Intercellular diffusion of a fluorescent sucrose analog via the septal junctions in a filamentous cyanobacterium. mBio 6:e02109-14. doi:10.1128/mBio.02109-14. 25784700 PMC4453526

[59] Bratbak G, Dundas I 1984 Bacterial dry matter content and biomass estimations. Appl Environ Microbiol 48:755–757. 6508285 10.1128/aem.48.4.755-757.1984 PMC241608

[60] Oelze J 2000 Respiratory protection of nitrogenase in *Azotobacter* species: is a widely held hypothesis unequivocally supported by experimental evidence? FEMS Microbiol Rev 24:321–333. doi:10.1111/j.1574-6976.2000.tb00545.x. 10978541

[61] Drozd J, Postgate JR 1970 Effects of oxygen on acetylene reduction, cytochrome content and respiratory activity of *Azotobacter chroococcum*. J Gen Microbiol 63:63–73. doi:10.1099/00221287-63-1-63. 5500027

[62] Foster RA, Kuypers MMM, Vagner T, Paerl RW, Musat N, Zehr JP 2011 Nitrogen fixation and transfer in open ocean diatom-cyanobacterial symbioses. ISME J 5:1484–1493. doi:10.1038/ismej.2011.26. 21451586 PMC3160684

[63] Hilton JA, Foster RA, James Tripp H, Carter BJ, Zehr JP, Villareal TA 2013 Genomic deletions disrupt nitrogen metabolism pathways of a cyanobacterial diatom symbiont. Nat Commun 4:1767. doi:10.1038/ncomms2748. 23612308 PMC3667715

[64] Farnelid H, Turk-Kubo K, Del Carmen Muñoz-Marín M, Zehr JP 2016 New insights into the ecology of the globally significant uncultured nitrogen-fixing symbiont UCYN-A. Aquat Microb Ecol 77:128–138.

[65] Eichner M, Kranz SA, Rost B 26 3 2014, posting date. Combined effects of different CO2 levels and N sources on the diazotrophic cyanobacterium Trichodesmium. Physiol Plant doi:10.1111/ppl.12172. PMC4260171 24547877

[66] Milligan AJ, Berman-Frank I, Gerchman Y, Dismukes GC, Falkowski PG 2007 Light-dependent oxygen consumption in nitrogen-fixing cyanobacteria plays a key role in nitrogenase protection. J Phycol 43:845–852. doi:10.1111/j.1529-8817.2007.00395.x.

[67] Mulholland MR, Capone DG 2000 The nitrogen physiology of the marine N_2_-fixing cyanobacteria *Trichodesmium* spp. Trends Plant Sci 5:148–153. doi:10.1016/S1360-1385(00)01576-4. 10740295

[68] Berman-Frank I, Lundgren P, Falkowski P 2003 Nitrogen fixation and photosynthetic oxygen evolution in cyanobacteria. Res Microbiol 154:157–164. doi:10.1016/S0923-2508(03)00029-9. 12706503

[69] Küpper H, Šetlík I, Seibert S, Prášil O, Šetlikova E, Strittmatter M, Levitan O, Lohscheider J, Adamska I, Berman-Frank I 2008 Iron limitation in the marine cyanobacterium *Trichodesmium* reveals new insights into regulation of photosynthesis and nitrogen fixation. New Phytol 179:784–798. doi:10.1111/j.1469-8137.2008.02497.x. 18513224

[70] Rodriguez IB, Ho T-Y 24 3 2014, posting date. Diel nitrogen fixation pattern of *Trichodesmium*: the interactive control of light and Ni. Sci Rep doi:10.1038/srep04445. PMC3963029 24658259

