# Supplementary material for: Mechanistic Model for the Coexistence of Nitrogen Fixation and Photosynthesis in Marine Trichodesmium
Source: mSystems. 2019 Aug 6;4(4):e00210-19. doi: 10.1128/mSystems.00210-19 (PMC6687940; doi:10.1128/mSystems.00210-19)
Supplement: TEXT S1 [file mSystems.00210-19-s0001.pdf]

Supplementary Methods

Here we describe some details of the model. A list of parameters with units is provided in Table S1 below. Also, values for fixed and adjustable parameters are summarized in Table S2 and S3 respectively.

Computing concentrations and fluxes of O_2. Under the pseudo-steady state, the time variation terms in [eq. 11] ~ [eq. 13] becomes zero. Thus,

$$0 = -J_{O2}^{PB} - J_{O2}^{PN} + \left(F_{Cfix} - F_{Res}^{P}\right)Y^{O2:C} \quad \text{[eq. S1]}$$

$$0 = J_{O2}^{PN} + J_{O2}^{BN} - F_{Res}^{N}Y^{O2:C} \quad \text{[eq. S2]}$$

$$0 = -J_{O2}^{BE} + J_{O2}^{PB} - J_{O2}^{BN} \quad \text{[eq. S3]}$$

since $J_{O2}^{ij} = A_{ij}\left([O_2]_i - [O_2]_j\right)$ with $i, j = P, N, B, E$, equation [eq. S1] ~ [eq. S3] are expanded to

$$0 = -A_{PB}([O_2]_P - [O_2]_B) - A_{PN}([O_2]_P - [O_2]_N) + \left(F_{Cfix} - F_{Res}^{P}\right)Y^{O2:C} \quad \text{[eq. S4]}$$

$$0 = A_{PN}([O_2]_P - [O_2]_N) + A_{BN}([O_2]_B - [O_2]_N) - F_{Res}^{N}Y^{O2:C} \quad \text{[eq. S5]}$$

$$0 = -A_{BE}([O_2]_B - [O_2]_E) + A_{PB}([O_2]_P - [O_2]_B) - A_{BN}([O_2]_B - [O_2]_N) \quad \text{[eq. S6]}$$

Given intracellular oxygen is small for non-photosynthetic cells ($[O_2]_N$ ~ 0) and O_2 in the environment ($[O_2]_E$) is constant, we solve [eq. S4]~[eq. S6] for $[O_2]_P$, $[O_2]_B$ and F_{Res}^{N};

$$[O_2]_P = \frac{aA_{PB} + aA_{BN} + aA_{BE} + A_{PB}A_{BE}[O_2]_E}{A_{PN}A_{PB} + A_{PN}A_{BN} + A_{PB}A_{BN} + A_{PN}A_{BE} + A_{PB}A_{BE}} \qquad \text{[eq. S7]}$$

$$[O_2]_B = \frac{aA_{PB} + A_{PN}A_{BE}[O_2]_E + A_{PB}A_{BE}[O_2]_E}{A_{PN}A_{PB} + A_{PN}A_{BN} + A_{PB}A_{BN} + A_{PN}A_{BE} + A_{PB}A_{BE}} \qquad \text{[eq. S8]}$$

$$F_{Res}^{N} = \frac{aA_{PN}A_{PB} + aA_{PN}A_{BN} + aA_{PB}A_{BN} + aA_{PN}A_{BE} + A_{PN}A_{PB}A_{BE}[O_2]_E + A_{PN}A_{BN}A_{BE}[O_2]_E + A_{PB}A_{BN}A_{BE}[O_2]_E}{Y^{O2:C}(A_{PN}A_{PB} + A_{PN}A_{BN} + A_{PB}A_{BN} + A_{PN}A_{BE} + A_{PB}A_{BE})} \qquad \text{[eq. S9]}$$

where $a = \left(F_{Cfix} - F_{Res}^{P}\right)Y^{O2:C}$. To test whether F_{Res}^{N} is supported by the maximum possible respiration rate based on available carbon storage

$$F_{Res}^{Nmax} = F_{Res}^{NmaxC} f_N \frac{C_{Sto}^{N}}{C_{Sto}^{N} + K_C} \qquad \text{[eq. S10]}$$

we compare F_{Res}^{N} and F_{Res}^{Nmax}. If F_{Res}^{N} is greater than F_{Res}^{Nmax}, we replace a value of F_{Res}^{N} with F_{Res}^{Nmax} and solve [eq. S4]~[eq. S6], this time, for $[O_2]_P$, $[O_2]_N$ and $[O_2]_B$:

$$\begin{aligned}[O_2]_P = (&aA_{PN}A_{PB} + aA_{PN}A_{BN} + aA_{PB}A_{BN} + aA_{PN}A_{BE} + aA_{BN}A_{BE} + A_{PN}A_{PB}b + A_{PN}A_{BN}b + A_{PB}A_{BN}b \\ &+A_{PN}A_{BE}b + A_{PN}A_{PB}A_{BE}[O_2]_E + A_{PN}A_{BN}A_{BE}[O_2]_E + A_{PB}A_{BN}A_{BE}[O_2]_E) \\ &/((A_{PN}A_{PB} + A_{PN}A_{BN} + A_{PB}A_{BN})A_{BE})\end{aligned} \qquad \text{[eq. S11]}$$

$$\begin{aligned}[O_2]_N = (&aA_{PN}A_{PB} + aA_{PN}A_{BN} + aA_{PB}A_{BN} + aA_{PN}A_{BE} + A_{PN}A_{PB}b + A_{PN}A_{BN}b + A_{PB}A_{BN}b \\ &+A_{PN}A_{BE}b + A_{PB}A_{BE}b + A_{PN}A_{PB}A_{BE}[O_2]_E + A_{PN}A_{BN}A_{BE}[O_2]_E + A_{PB}A_{BN}A_{BE}[O_2]_E) \\ &/((A_{PN}A_{PB} + A_{PN}A_{BN} + A_{PB}A_{BN})A_{BE})\end{aligned} \qquad \text{[eq. S12]}$$

$$[O_2]_B = \frac{a + b + A_{BE} + [O_2]_E}{A_{BE}} \qquad \text{[eq. S13]}$$

where $b = -F_{Res}^{N}Y^{O2:C}$. The solution for $[O_2]_N$ is greater than zero, since respiratory protection is not sufficient due to a shortage of carbon supply.

Computed F_{Res}^{N} can be separated into two parts, respiration providing energy for N_2 fixation F_{ResN2}, and respiratory protection F_{RP}:

$$F_{Res}^{N} = F_{ResN2} + F_{RP} \qquad \text{[eq. S14]}$$

F_{ResN2} is proportional to the rate of N_2 fixation:

$$F_{ResN2} = F_{Nfix}Y_{Res}^{C:N} \qquad \text{[eq. S15]}$$

where $Y_{Res}^{C:N}$ is obtained based on the energetic balance (1–3). F_{RP} is obtained from [eq. S14] with F_{Res}^{N} and F_{ResN2}.

In this model, we use s^{-1} for the unit of a diffusion coefficient A_{ij}. To convert it to a widely used unit (m^2 s^{-1}), we used a typical size of *Trichodesmium*; the conversion is done based on a typical size (diameter of 9.670 µm and length of 554.145 µm) and membrane thickness (0.076 µm) of *Trichodesmium*. Diameter and membrane thickness are estimated from microscopic images of cross sections of *Trichodesmium* (4). To estimate the total length of a trichome, the length of one cell is estimated from a microscopic image of *Trichodesmium* (9.23575 µm) (5), and we multiplied this number by 60 a typical number of cells in a trichome (5).

We assumed a cylinder for the shape of a trichome and diffusive flux (normalized by volume) into the cell from the surface of the membrane can be described as follows (6):

$$J_{O2} = \frac{-2\pi D_{O2}\varepsilon_m L}{V}\left(\ln\left(\frac{R}{R+L_g}\right)\right)^{-1}([O_2]_{out} - [O_2]_{in}) \quad \text{[eq. S16]}$$

where D_{O2} is the diffusivity of water ε_m is the diffusivity of the membrane layer relative to water, L is the length of a part of cylinder of which we estimate the flux (either the total length of photosynthetic cells or non-photosynthetic cells), V is the total volume of the trichome, R is the radius of the cytoplasmic space, L_g is the thickness of the cell membrane layer, and $[O_2]_{out}$ and $[O_2]_{in}$ are O_2 concentration right outside/inside of the membrane, respectively. Our model is a simplified version of [eq. S16] with a diffusion coefficient of A:

$$A = \frac{-2\pi D_{O2}\varepsilon_m L}{V}\left(\ln\left(\frac{R}{R+L_g}\right)\right)^{-1} \quad \text{[eq. S17]}$$

and this equation is used for the conversion between A and $D_{O2}\varepsilon_m$ for fluxes between cells and boundary layers. Here we note that the units of both sides are s^{-1}.

The O_2 flux from photosynthetic cells to non-photosynthetic cells (normalized by V) is represented as 1D diffusion:

$$J_{O2}^{PN} = \frac{2\pi R^2 D_{O2}\varepsilon_m}{VL_g}([O_2]_P - [O_2]_N) \quad \text{[eq. S18]}$$

which assumes typical 2 diazocytes per trichome (5) with double cellular membranes between cells. Our model simplifies [eq. S18] with

$$A_{PN} = \frac{2\pi R^2 D_{O2}\varepsilon_m}{VL_g} \quad \text{[eq. S19]}$$

and it is used for the conversion between A_{PN} and $D_{O2}\varepsilon_m$. The units of both sides are s^{-1}.

References

1. **Rittmann BE, McCarty PL**. 2001. Environmental Biotechnology: Principles and Applications. McGraw-Hill: New York, NY.
2. **Inomura K, Bragg J, Follows MJ**. 2017. A quantitative analysis of the direct and indirect costs of nitrogen fixation: a model based on *Azotobacter vinelandii*. ISME J **11**:166–175.
3. **Inomura K, Bragg J, Riemann L, Follows MJ**. 2018. A quantitative model of nitrogen fixation in the presence of ammonium. PLOS ONE **13**:e0208282. https://doi.org/10.1371/journal.
4. **Carpenter EJ, O'Neil JM, Dawson R, Capone DG, Siddiqui PJA, Roenneberg T, Bergman B**. 1993. The tropical diazotrophic phytoplankter *Trichodesmium*: biological characteristics of two common species. Mar Ecol Prog Ser **95**:295–304.
5. **El-Shehawy R, Lugomela C, Ernst A, Bergman B**. 2003. Diurnal expression of *hetR* and diazocyte development in the filamentous non-heterocystous cyanobacterium *Trichodesmium erythraeum*. Microbiology2 **149**:1139–1146.
6. **Staal M, Meysman FJR, Stal LJ**. 2003. Temperature excludes N_2-fixing heterocystous cyanobacteria in the tropical oceans. Nature **425**:504–507.
