# Supplementary material for: Mechanistic Model for the Coexistence of Nitrogen Fixation and Photosynthesis in Marine Trichodesmium
Source: mSystems. 2019 Aug 6;4(4):e00210-19. doi: 10.1128/mSystems.00210-19 (PMC6687940; doi:10.1128/mSystems.00210-19)
Supplement: FIG S1 [file mSystems.00210-19-sf001.pdf]

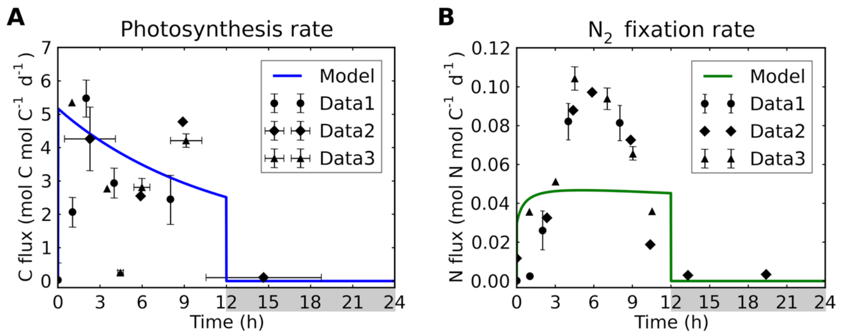

References

1. **Finzi-hart JA, Pett-Ridge J, Weber PK, Popa R, Fallon SJ, Gunderson T, Hutcheon ID, Nealson KH, Capone DG**. 2009. Fixation and fate of C and N in the cyanobacterium *Trichodesmium* using nanometer-scale secondary ion mass spectrometry. Proc Natl Acad Sci **106**:6345–6350.

2. **Berman-Frank I, Lundgren P, Chen Y-B, Küpper H, Kolber Z, Bergman B, Falkowski P**. 2001. Segregation of nitrogen fixation and oxygenic photosynthesis in the marine cyanobacterium *Trichodesmium*. Science **294**:1534–1537.
