# Supplementary figures and images for: Mechanistic Model for the Coexistence of Nitrogen Fixation and Photosynthesis in Marine Trichodesmium
Source: mSystems. 2019 Aug 6;4(4):e00210-19. doi: 10.1128/mSystems.00210-19 (PMC6687940; doi:10.1128/mSystems.00210-19)

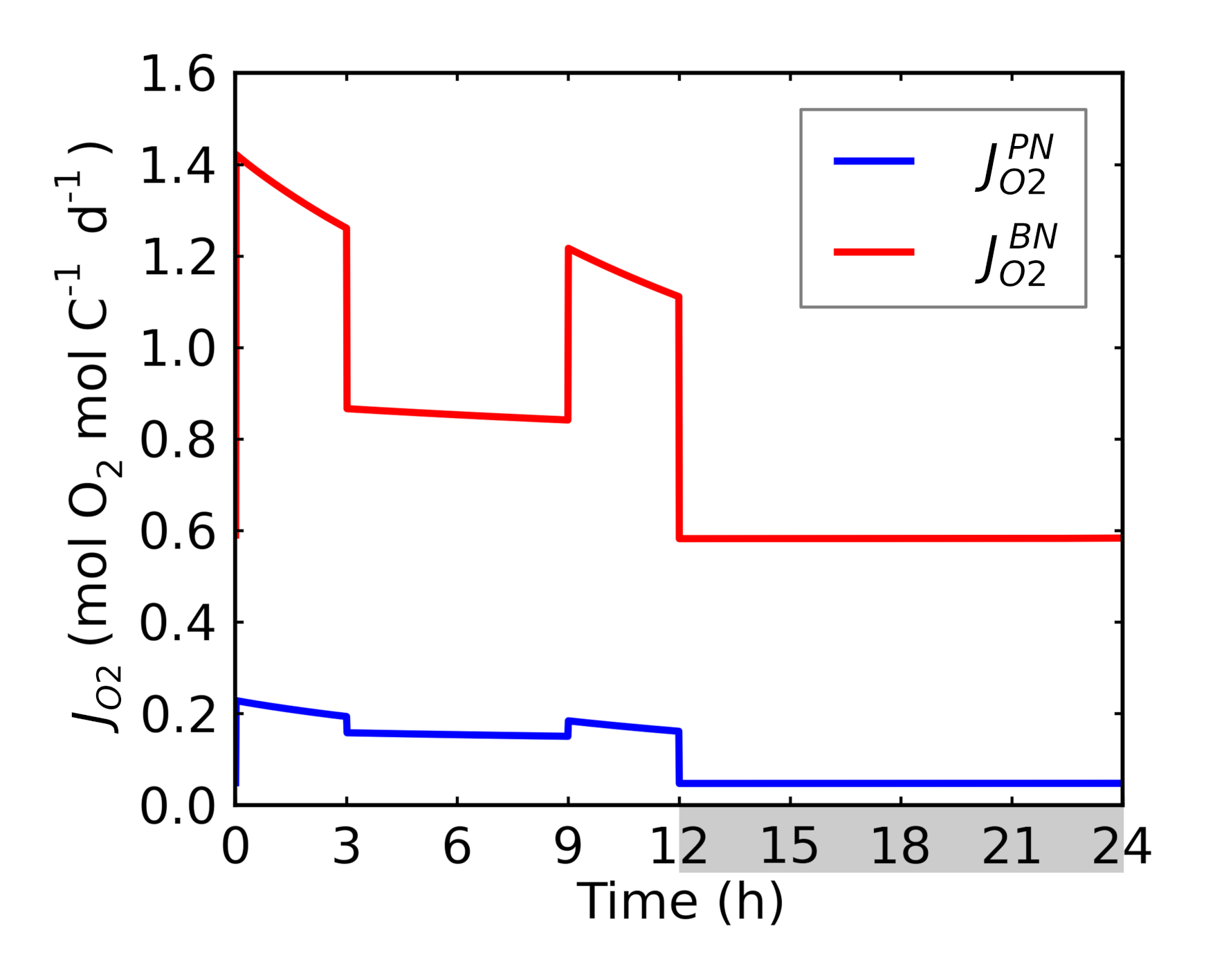

Supplement: FIG S2 [file mSystems.00210-19-sf002.tif]
