# Supplementary material for: Mechanistic Model for the Coexistence of Nitrogen Fixation and Photosynthesis in Marine Trichodesmium
Source: mSystems. 2019 Aug 6;4(4):e00210-19. doi: 10.1128/mSystems.00210-19 (PMC6687940; doi:10.1128/mSystems.00210-19)
Supplement: TABLE S1 [file mSystems.00210-19-st001.pdf]

Parameter	Unit	Definition
F_{Nfix}	mol m^{-3} s^{-1}	N_2 fixation rate
F_{Nfix}^{max}	mol m^{-3} s^{-1}	Maximum N_2 fixation rate
$[O_2]_P$	mol m^{-3}	O_2 concentration in photosynthetic cells (*P*)
$[O_2]_N$	mol m^{-3}	O_2 concentration in non-photosynthetic cells (*N*)
$[O_2]_B$	mol m^{-3}	O_2 concentration in boundary layers (*B*)
$[O_2]_E$	mol m^{-3}	O_2 concentration in the environment (*E*)
$[O_2]_{crit}$	mol m^{-3}	Critical oxygen concentration
C_{Sto}	mol m^{-3}	C storage
C_{Sto}^{N}	mol m^{-3}	C storage per total volume of non-photosynthetic cells in a trichome
C_{Sto}^{max}	mol m^{-3}	maximum C storage
K_C	mol m^{-3}	Half saturation constant of C storage
N_{Sto}	mol m^{-3}	N storage
N_{Sto}^{max}	mol m^{-3}	Maximum N storage
K_N	mol m^{-3}	Half saturation constant of N storage
t	s	Time
F_{Cfix}	mol m^{-3} s^{-1}	C fixation rate
F_{Bio}	mol m^{-3} s^{-1}	Biomass production rate
$Y_{Nfix}^{C:N}$	mol mol^{-1}	C:N of N_2 fixation
F_{Res}	mol m^{-3} s^{-1}	Respiration rate
$Y_{Bio}^{N:C}$	mol mol^{-1}	N:C of biomass
V_P	m^3	Total volume of photosynthetic cells in a trichome
V_N	m^3	Total volume of non-photosynthetic cells in a trichome
V_B	m^3	Volume of boundary layers
V	m^3	Volume of a trichome
J_{O2}^{ij}	mol m^{-3} s^{-1}	O_2 flux from *i* to *j*. *i*, *j* = *P*, *N*, *B*, *E*
$Y^{O2:C}$	mol O_2 mol C^{-1}	O_2:C in respiration and photosynthesis
F_{Res}^{P}	mol m^{-3} s^{-1}	Respiration rate in photosynthetic cells
F_{Res}^{N}	mol m^{-3} s^{-1}	Respiration rate in non-photosynthetic cells
F_{Bio}^{max}	mol m^{-3} s^{-1}	Maximum biomass production rate
F_{Cfix}^{Chl}	mol m^{-3} s^{-1} mol^{-1}	C fixation rate per chlorophyll C
$F_{CfixMax}^{Chl}$	mol m^{-3} s^{-1} mol^{-1}	Maximum C fixation rate per chlorophyll C

K_I	μ mol^{-1} m^2 s	Light saturation coefficient
I	μ mol m^{-2} s^{-1}	Light intensity
f_P	dimensionless	Fraction of photosynthetic cells
f_N	dimensionless	Fraction of non-photosynthetic cells
$f_{NITROGE}$	dimensionless	Fraction of cells with nitrogenase among non-photosynthetic cells
Chl_P	mol m^{-3}	Chlorophyll in photosynthetic cells
Chl_{full}	mol m^{-3}	Chlorophyll in a trichome
$Y^{Res:Bio}$	mol mol^{-1}	Respiration for biomass production : Biomass production
μ_{max}	s^{-1}	Maximum growth rate
F_{Cfix}^{Qc}	s^{-1}	C fixation rate per cellular carbon quota
$Y^{Bio:Cfix}$	mol mol^{-1}	Biomass yield of production
Q_C	mol m^{-3}	Cellular C quota
F_{RP}	mol m^{-3} s^{-1}	Respiratory protection
F_{ResN2}	mol m^{-3} s^{-1}	Respiration for N_2 fixation
$Y^{Res:Nfix}$	mol mol^{-1}	Respiration for N_2 fixation: C consumption for N_2 fixation
J_{O2}	mol m^{-3} s^{-1}	O_2 flux between cells and boundary layer
D_{O2}	m^2 s^{-1}	Diffusion coefficient of water
ε_m	dimensionless	Diffusion coefficient of cell membrane layers : diffusion coefficient of water
L	m	Length of a cylinder for J_{O2}
R	m	Radius of cytoplasm
L_g	m	Thickness of the cell membrane layers
$[O_2]_{out}$	mol m^{-3}	Oxygen concentration right outside of the cell membrane
$[O_2]_{in}$	mol m^{-3}	Oxygen concentration of cytoplasm
A	s^{-1}	Diffusion coefficient of oxygen through cell membrane layers

The volume of a trichome is used for normalization unless stated otherwise.
