# Supplementary material for: Mechanistic Model for the Coexistence of Nitrogen Fixation and Photosynthesis in Marine Trichodesmium
Source: mSystems. 2019 Aug 6;4(4):e00210-19. doi: 10.1128/mSystems.00210-19 (PMC6687940; doi:10.1128/mSystems.00210-19)
Supplement: TABLE S2 [file mSystems.00210-19-st002.pdf]

Parameter	Value	Unit	Explanations
$[O_2]_E$	0.213	mol O_2 m^{-3}	Saturated concentration at 25 ˚C and salinity of 34 (1).
$Y_{Nfix}^{C:N}$	1	mol mol^{-1}	(2)(3).
$Y_{Bio}^{N:C}$	0.159	mol mol^{-1}	(4).
I	400	µmol m^{-2} s^{-1}	Boundary condition.
$Y^{Res:Bio}$	0.4	mol mol^{-1}	Based on energetic balance (5)(2) with energy transfer efficiency of 0.6 (5) With biomass stoichiometry of $C_5H_7O_2N_{0.8}$.
$Y^{Res:Nfix}$	1.04	mol mol^{-1}	
Q_C	18333	mol m^{-3}	(6).

The parameters are listed roughly in order of appearance in Methods and Supplementary Methods.

References

1. **Benson BB, Krause D**. 1984. The concentration and isotopic fractionation of oxygen dissolved in freshwater and seawater in equilibrium with the atmosphere. Limnol Oceanogr **29**:620–632.

2. **Inomura K, Bragg J, Follows MJ**. 2017. A quantitative analysis of the direct and indirect costs of nitrogen fixation: a model based on *Azotobacter vinelandii*. ISME J **11**:166–175.

3. **Inomura K, Bragg J, Riemann L, Follows MJ**. 2018. A quantitative model of nitrogen fixation in the presence of ammonium. PLOS ONE **13**:e0208282. https://doi.org/10.1371/journal.

4. **LaRoche J, Breitbarth E**. 2005. Importance of the diazotrophs as a source of new nitrogen in the ocean. J Sea Res **53**:67–91.

5. **Rittmann BE, McCarty PL**. 2001. Environmental Biotechnology: Principles and Applications. McGraw-Hill: New York, NY.

6. **Bratbak G, Dundas I**. 1984. Bacterial dry matter content and biomass estimations. Appl Environ Microbiol **48**:755–757.
