# Supplementary material for: Mechanistic Model for the Coexistence of Nitrogen Fixation and Photosynthesis in Marine Trichodesmium
Source: mSystems. 2019 Aug 6;4(4):e00210-19. doi: 10.1128/mSystems.00210-19 (PMC6687940; doi:10.1128/mSystems.00210-19)
Supplement: TABLE S3 [file mSystems.00210-19-st003.pdf]

Parameter	Value	Unit
f_N	0.2 (0.45 $t = 3 \sim 9$ h)	dimensionless
F_{Nfix}^{full}	0.21	mol m^{-3} s^{-1}
$[O_2]_{crit}$	0.1	mol O_2 m^{-3}
K_C	3667	mol m^{-3}
$f_{NITROGE}$	0.4	dimensionless
C_{Sto} ($t = 0$)	1000	mol m^{-3}
N_{Sto} ($t = 0$)	1000	mol m^{-3}
A_{PB}	$3.6 \times f_P$	s^{-1}
A_{PN}	0.062	s^{-1}
A_{BN}	$3.6 \times f_N$	s^{-1}
A_{BE}	3.6	s^{-1}
F_{Bio}^{max}	0.053	mol m^{-3} s^{-1}
$F_{CfixMax}^{Chl}$	0.00268	mol mol C^{-1} s^{-1}
K_I	0.01	µmol m^{-2} s^{-1}
Chl_{full}	535	mol C m^{-3}
C_{Sto}^{max}	36666	mol m^{-3}
F_{Res}^{NmaxC}	10.6	mol m^{-3} s^{-1}

The parameters are listed roughly in order of appearance in Methods and Supplementary Methods.
